# Strengthening antimicrobial resistance policies: lessons from the design and implementation phase of the EU-JAMRAI 2 Sustainability Guidance Tool

**DOI:** 10.5588/pha.25.0045

**Published:** 2026-03-06

**Authors:** B. Davido, M. Kharkhordine, D. Merillon, M. Fuentes-Braesch

**Affiliations:** 1 Maladies Infectieuses, Hôpital Raymond-Poincaré, Université Paris Saclay, AP-HP, Garches, France;; 2 UMR1173, Université Versailles Saint-Quentin, Montigny-Le-Bretonneux, France;; 3 Mission Ministérielle de la Prévention des infections et de l’antibiorésistance, Direction Générale de la Santé, Paris, France.

**Keywords:** antimicrobial resistance, sustainability, joint action, embedding, policy integration, One Health

## Abstract

Antimicrobial resistance (AMR) threatens global health and requires sustained, integrated responses beyond short-term initiatives. EU-JAMRAI 2 mobilises over 120 partners to produce scalable solutions aligned with One Health priorities. To ensure long-term uptake, the French Ministry of Health developed a Sustainability Guidance Tool (SGT) supporting work packages in defining priority outcomes, anticipating risks, and embedding results into national systems. This dynamic approach, based on iterative revision and alignment with National Action Plans (NAPs), strengthens governance, financing, monitoring, and policy integration. By summarising early lessons from design and implementation, this article informs future transnational AMR policy planning and sustainability strategies.

Antimicrobial resistance (AMR) is a growing global health threat, ranked among the top three public health threats. Harder-to-treat infections increase complications and burden both patients and health systems. A recent global modelling study projects that over 39 million deaths could result from bacterial AMR between 2025 and 2050.^1^ Many countries have adopted National Action Plans (NAPs) grounded in a One Health approach. The European Joint Action on AMR and Healthcare-Associated Infections 2 (EU-JAMRAI 2), running from 2024 to 2027, unites over 120 partners across 30 countries. Supported by the WHO, FAO, WOAH, UNEP, ECDC, and EFSA, and co-funded under the EU4Health programme (€50 million), it aims to produce actionable, scalable AMR solutions. Given the unprecedent collective efforts, ensuring sustainability beyond the project duration is essential, defined as long-term uptake and implementation of priority outcomes within Member States and associated countries (MS/AC).

To mitigate unsustainable outcomes, the French Ministry of Health developed the Sustainability Guidance Tool (SGT).^2^ This peer-reviewed document supports technical thematic work packages (antimicrobial stewardship, infection prevention and control [IPC], surveillance, access to antibiotics, and awareness) in embedding their outputs into national systems for lasting improvements in policy, practice, and health outcomes.

This article summarises early lessons from SGT design and initial implementation to inform future AMR policy planning and sustainability strategies across countries.

## METHODS

This short communication is based on a narrative and conceptual synthesis of lessons learned from the design and early implementation of the SGT, drawing on

- Structured consultations with EU-JAMRAI 2 work package leaders,
- Iterative application of the SGT across thematic work packages,
- Synthesis of early implementation feedback coordinated by the French Ministry of Health.

Given the ongoing nature of the Joint Action, no formal outcome or impact evaluation is presented.

### Key principles for a sustainable action

The ST is built on four core principles that guide planning, implementation, evaluation, and integration of results into existing systems:

- Activities must address AMR needs, gaps, capacities, and regulatory contexts; overly generic approaches risk failure.
- Continuous stakeholder engagement across human, animal, environmental, diagnostic, regulatory, and policy sectors is essential to ensure ownership, accountability, feasibility, and alignment with real-world priorities.
- Coherence with existing NAPs and One Health programmes is crucial to avoid duplication and promote complementarity and alignment.
- Sustainability requires embedding governance, financing, monitoring and evaluation, network maintenance, and future updates to ensure lasting results.

### How the tool works in practice

**FIGURE 1.**
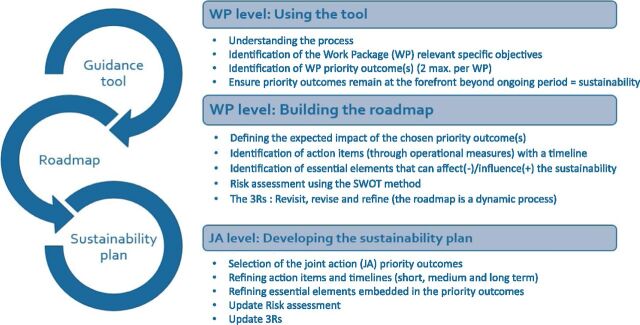
Operational workflow of the Sustainability Guidance Tool (SGT). This diagram illustrates the three-step approach used within each Work Package (WP): i) Using the tool to define specific objectives and priority outcomes; ii) Building the roadmap by identifying action items, essential elements, timelines, and risks through SWOT analysis; and iii) Developing the sustainability plan at Joint Action (JA) level through refinement of actions, timelines, and risk assessments. The process is iterative, applying the 3Rs (Revisit, Revise, Refine) to ensure continuous adaptation and long-term integration of priority outcomes.

Technical experts define one or more priority outcomes summarising their outputs, intended to persist beyond the project. Examples include implementing artificial intelligence for improved antibiotic prescribing, strengthening AMR communication channels, or establishing integrated surveillance reporting in specific regions. For each outcome, work packages set intermediate milestones, assess risks (e.g., staff turnover, funding gaps, and regulatory changes), and propose mitigation strategies informed by SWOT analysis.^3^ These are compiled into a priority outcomes table, enabling linkage to NAPs or policies to ensure relevance, legitimacy, and long-term uptake based on S.M.A.R.T. criteria.^4^ Figure 1 shows the relationship between guidance tool, roadmap, and sustainability plan.

### Periodic review: the 3Rs

Rather than following a fixed roadmap, work packages use an iterative process: Revisit priorities regularly to maintain relevance with evolving needs; Revise roles, resource allocations, or timelines in response to shifting conditions; Refine strategies and pathways based on new evidence.

This fosters adaptability, learning, and responsiveness. For instance, slower-than-expected network growth can prompt updates to the priority outcome table, such as adding training or reallocating resources before gaps emerge.

### Mapping what sustains the work

Sustainability is multidimensional, linking governance, resources, stakeholders, and timing of action across different system levels. Failure in any dimension can weaken the intervention. All priority outcomes collectively form the sustainability report, guiding long-term integration into EU policies.

### Embedding into NAPs and/or policies

Work packages aim for outputs to become self-evident foundations for improved practice. Some components (e.g., protocols or tools) may be one-off; others (e.g., training, reporting, and practice updates) require ongoing support. Teams estimate resources and identify formal mechanisms (agreements, regulations, budget lines, and political support) to institutionalise their work. Preparing policy briefs is strongly encouraged to assist policymakers in aligning decisions with JAMRAI 2 outcomes.

## DISCUSSION

**FIGURE 2.**
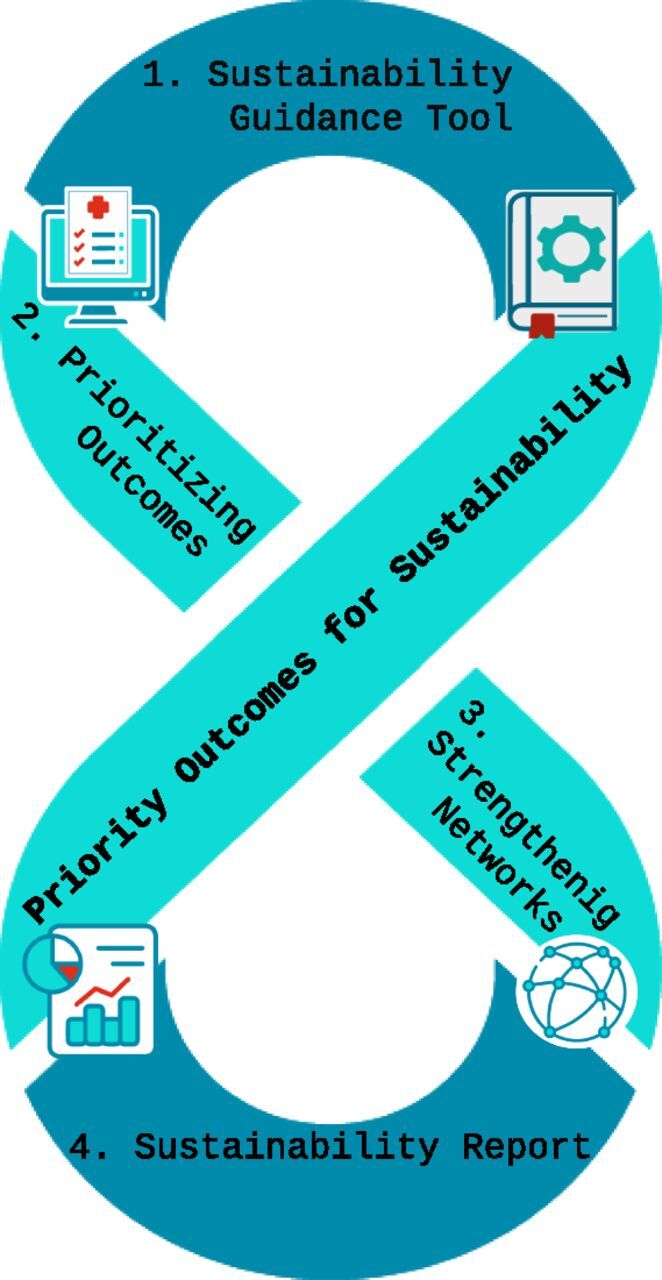
Core pillars of EU-JAMRAI 2 sustainability strategy. This diagram summarises the four foundational elements of EU-JAMRAI 2’s sustainability approach: i) the Sustainability Guidance Tool (SGT), which helps technical work streams design and embed outcomes; ii) prioritisation of outcomes for integration into National Action Plans or policy; iii) strengthening of EU and pan-EU networks to support maintenance, knowledge sharing, and dialogue; and iv) the Sustainability Report, which documents progress, extracts lessons, and formulates plans for further integration at national and EU levels (adapted from the EU-JAMRAI 2 Sustainability Guidance Tool, WP4, French Ministry of Health, January 2025).

SGT implementation highlights several key lessons. First, structured identification of priority outcomes and early risk assessment facilitates integration into national systems. Second, iterative application and regular revisiting of priorities (3Rs) support adaptability and embedment across work packages. Third, stakeholder engagement and alignment with NAPs enhance legitimacy and feasibility. Finally, contextual factors such as governance, funding, and political cycles influence sustainability and should be considered in future implementation. These lessons are summarised in Figure 2, illustrating the four core pillars of the EU-JAMRAI 2 sustainability approach: SGT, prioritisation of outcomes, network strengthening, and Sustainability Report.

Performance benchmarking is essential for sustaining AMR actions. The AMR accountability index^5^ provides a structured approach to compare national performance across multiple domains, including governance, financing, antimicrobial stewardship, IPC, and surveillance. However, these benchmarking metrics are meaningful only if the underlying systems are well-designed, adequately resourced, and capable of sustaining routine operations. The SGT contributes to this foundation by embedding priority outcomes into national systems, anticipating risks, and applying iterative refinement (3Rs), enhancing reliable cross-country AMR programme effectiveness.

While the WHO Implementation Handbook^6^ provides national-level guidance, it does not explicitly guide work streams to embed outputs into national systems or anticipate sustainability risks iteratively. By linking priority outcomes to NAPs and facilitating early risk assessment, the SGT fills this gap. Two complementary existing frameworks illustrate related but distinct approaches:

- FAO AMR Governance Frameworks^7^ provide guidance for implementing AMR NAPs specifically in the food and agriculture sectors, highlighting sector-focused priority setting and resource mobilisation.
- EU-JAMRAI 1 sustainability report^8^ identified key areas (surveillance, stewardship, IPC) but lacked structured tools for interactive prioritisation, risk anticipation, and systematic NAP alignment.

By combining macro-level guidance with an operational tool, SGT supports embedding technical outputs into routine systems aligned with NAPs and One Health priorities.

Limitations to this approach include: SGT is implemented in heterogeneous contexts with variability in governance, resources, and institutional capacity. Funding constraints and political cycles may also influence the long-term sustainability. Future work should assess how these contextual factors affect the SGT effectiveness and transferability across different settings.

The expected pathway of the SGT can be summarised as follows: SGT implementation → stronger system integration of AMR actions → sustained antimicrobial stewardship, IPC, and surveillance activities → improved public health outcomes related to AMR. While these benefits are conceptually expected, they have not yet been formally measured.

### Key takeaways for policy and practice

Structured, iterative approaches combined with stakeholder engagement and alignment with NAPs enhance long-term sustainability of AMR actions. Early lessons, performance benchmarking, comparison with WHO guidance, and contextual understanding provide a practical framework for other transnational AMR initiatives.

## CONCLUSION

The SGT provides a structured framework to support the long-term integration of AMR actions, which will require further empirical assessment as the joint action progresses.

## References

[1] Naghavi M, Global burden of bacterial antimicrobial resistance 1990–2021: a systematic analysis with forecasts to 2050. Lancet. 2024;404(10459):1199-1226. 39299261 10.1016/S0140-6736(24)01867-1 PMC11718157

[2] EU JAMRAI 2 Consortium. EU-JAMRAI 2: our approach to sustainability, 2025. https://eu-jamrai.eu/sustainability/.

[3] Puyt RW, Lie FB, Wilderom CPM. The origins of SWOT analysis. Long Range Plan. 2023;56(3):102304.

[4] Doran GT. There’s a S.M.A.R.T. way to write management’s goals and objectives. Manag Rev. 1981;70:35-36.

[5] Anderson M, Promoting sustainable national action to tackle antimicrobial resistance: a proposal to develop an antimicrobial resistance accountability index. Lancet Microbe. 2024;5(11):100997. 39341218 10.1016/j.lanmic.2024.100997

[6] World Health Organization. WHO implementation handbook for national action plans on antimicrobial resistance: guidance for the human health sector. Geneva: WHO, 2022.

[7] Food and Agriculture Organization of the United Nations. Methodology to analyse AMR-relevant legislation in the food and agriculture sector. Rome, Italy: FAO, 2020.

[8] Pulcini C, Bouqueau M. European Union Joint Action (JA) on Antimicrobial Resistance and Healthcare-Associated Infections (EU-JAMRAI). Report on sustainability plan, 2021. https://eu-jamrai.eu/wp-content/uploads/2021/03/EUjamrai_D4.4_Report-on-sustainability-plan_WP4_MoH_20210225.pdf.

